# Edible Alien Invasive Plants: Ecosystem Services for Health Benefits?

**DOI:** 10.3390/plants15213344

**Published:** 2026-10-28

**Authors:** Ekaterina Kozuharova, Ivan Stambolov, Irena Mincheva, Aleksandar Shkondrov, Ilina Krasteva, Francesco M. Raimondo, Giuseppe Antonio Malfa, Rosaria Acquaviva, Vladimir Vladimirov, Vivienne Spadaro

**Affiliations:** 1 Department of Pharmacognosy, Faculty of Pharmacy, Medical University-Sofia, 1000 Sofia, Bulgaria; istambolov@pharmfac.mu-sofia.bg (I.S.); shkondrov@pharmfac.mu-sofia.bg (A.S.); ikrasteva@pharmfac.mu-sofia.bg (I.K.); 2 Institute of Chemical Engineering, Bulgarian Academy of Sciences, Acad. Georgi Bonchev Str., Bl. 103, 1113 Sofia, Bulgaria; irenamincheva@gmail.com; 3 PLANTA/Center for Research, Documentation and Training, Via Serraglio Vecchio 28, 90123 Palermo, Italy; raimondo@centroplantapalermo.org; 4 Department of Drug and Health Sciences, University of Catania, Viale A. Doria 6, 95125 Catania, Italy; gmalfa@unict.it (G.A.M.); racquavi@unict.it (R.A.); 5 Research Centre on Nutraceuticals and Health Products (CERNUT), University of Catania, Viale A. Doria 6, 95125 Catania, Italy; 6 Institute of Biodiversity and Ecosystem Research-Bulgarian Academy of Sciences (IBER-BAS), Acag. G. Bonchev Str. 23, 1113 Sofia, Bulgaria; vladimir_dv@abv.bg; 7 Department of Biological, Chemical and Pharmaceutical Sciences and Technologies, Section of Botany, Anthropology and Zoology, University of Palermo, Via Archirafi 38, 90123 Palermo, Italy

**Keywords:** biological invasions, ethnobotany, bioactive compounds, nutraceutical potential, sustainable management

## Abstract

Invasive alien plants present severe global ecological challenges by disrupting native ecosystem equilibriums and threatening local biodiversity. However, many aggressive species possess extensive histories of traditional edibility and accumulate complex secondary metabolites, making them promising candidates for sustainable utilization. This comprehensive review evaluates six prominent edible invasive alien plant taxa—*Amaranthus hybridus*, *Reynoutria × bohemica*, *Opuntia humifusa*, *Oxalis corniculata*, *Phytolacca americana*, and *Carpobrotus edulis*—to assess their potential integration into risk-aware management frameworks. Following PRISMA guidelines, a comprehensive literature review was conducted across major databases (1990–2026) to summarize their distribution, ethnobotanical consumption practices, phytochemical profiles, preclinical bioactivities, and safety limits. The results show that these plants serve as vital nutritional or culinary resources globally and contain valuable bioactive compounds, including flavonoids, anthraquinones, stilbenes, and triterpenoids. Preclinical evaluations demonstrate potent anti-diabetic, anti-inflammatory, antioxidant, and antiproliferative properties. However, exploitation requires a strict evaluation of toxicity issues, such as the high calcium oxalate levels in *Oxalis corniculata* or high triterpenoid saponin content in *Phytolacca americana*. In conclusion, managing these species within a risk-aware framework transforms invasive concerns into valuable bioresources, supporting sustainable development and human health.

## 1. Introduction

Ecosystem services are defined as direct and indirect contributions of ecosystems to human well-being, encompassing the ecological processes and natural resources upon which human societies depend [1]. The Millennium Ecosystem Assessment (MA) has established a widely adopted classification of ecosystem services into four categories: provisioning services, including food, freshwater, and raw materials; regulating services, such as climate regulation, pollination, erosion control, and water purification; cultural services, including recreational, esthetic, and spiritual benefits; and supporting services, such as soil formation, nutrient cycling, and primary production, which underpin the other service categories [1]. These services are closely interconnected, and changes in ecosystem structure and functioning may generate both beneficial and adverse consequences for human well-being.

Global food insecurity and the growing demand for sustainable, nutritious food highlight the need to identify and develop new plant-based food resources. Exploring underutilized and wild edible plants may help diversify food sources and strengthen food-system resilience. Recent reports from several countries highlight the widespread and regular consumption of wild food plants, predominantly among rural and indigenous populations, though increasingly observed in urban environments as well and sometimes with economic importance [2,3,4,5,6,7]. A manifestation of the growing demand for sustainable and diverse food systems is the Slow Food movement, which recognizes the value of wild edible plants and their traditional uses in local food cultures, advocating for the preservation of food biodiversity and the knowledge associated with gathering, preparing, and consuming these resources [8].

Invasive alien plants present global ecological challenges. They heavily disrupt native ecosystem equilibriums [9]. Biological invasions can substantially alter the provision of ecosystem services, creating complex interactions between ecological impacts and socioeconomic benefits. Invasive alien species may impair ecosystem functioning, reduce native biodiversity, and compromise services upon which local communities depend. However, these invasive plants may offer unique biological resources including food, medicinal products, fodder, fuel, and raw materials, while contributing to selected regulating or cultural services [10,11,12]. Many invasive species are traditionally edible. They contain valuable bioactive secondary compounds [13]. Utilizing these species can support sustainable management [14,15,16,17], though this approach requires strict safety and risk-aware assessment frameworks regarding bioactive compounds. In relation to that, a comprehensive evaluation of invasive plant species begins with assessing their traditional applications within their native habitats [18,19,20].

The aim of this study is to provide an overview of six known as edible invasive alien plants, namely *Amaranthus hybridus* L., *Reynoutria* × *bohemica* Chrtek & Chrtková (syn. *Fallopia × bohemica* (Chrtek & Chrtková) J.P.Bailey), *Opuntia humifusa* (Raf.) Raf., *Oxalis corniculata* L., *Phytolacca americana* L., and *Carpobrotus edulis* (L.) N.E.Br., and to examine their secondary metabolites regarding their pharmacological effects including benefits and toxicity.

These six specific plant taxa, chosen for this comprehensive review, were selected for three interconnected reasons:

1. Severe threats to biodiversity: They grow rapidly and densely, outcompeting native local flora in natural environments like riversides, grasslands, and rocky/sandy zones. Particularly species in this selection, such as *O. humifusa* and *R. × bohemica*, are explicitly cited as being among the top ten worst aggressive invasive plants [9].
2. Rich and well-documented traditional edibility: Rather than being obscure weeds, these plants have extensive histories of human consumption in their native ranges and even newly invaded territories. For example, serving as a traditional leafy green (“quelite” or “marog”), *A. hybridus* supplements nutritional profiles in diets across its native range Latin America [21] and in newly invaded Africa [22,23]. *P. americana* is also an example of a dynamic culinary integration in newly invaded territories [24]. Although *C. edulis* is a particularly well-established culinary delicacy and is locally traded as food in its native South African range, its edible potential is also being recognized outside its native range, including in Portugal [25,26].
3. Untapped or promising pharmacological value (nutraceutical potential): These six plants accumulate high concentrations of complex secondary metabolites—such as flavonoids, anthraquinones, stilbenes, and triterpenoids. They show significant preclinical (in vitro and in vivo) bioactivities, such as potent anti-diabetic (including α-glucosidase inhibition), antioxidant, and anti-inflammatory properties. At the same time, it is crucial to address toxicity issues, such as the high calcium oxalate levels in *O. corniculata* or the high triterpenoid saponin content in *P. americana*.

## 2. Results and Discussion

### 2.1. Distribution and Ecological Specifics

#### 2.1.1. *Amaranthus hybridus*

**Figure 1:**
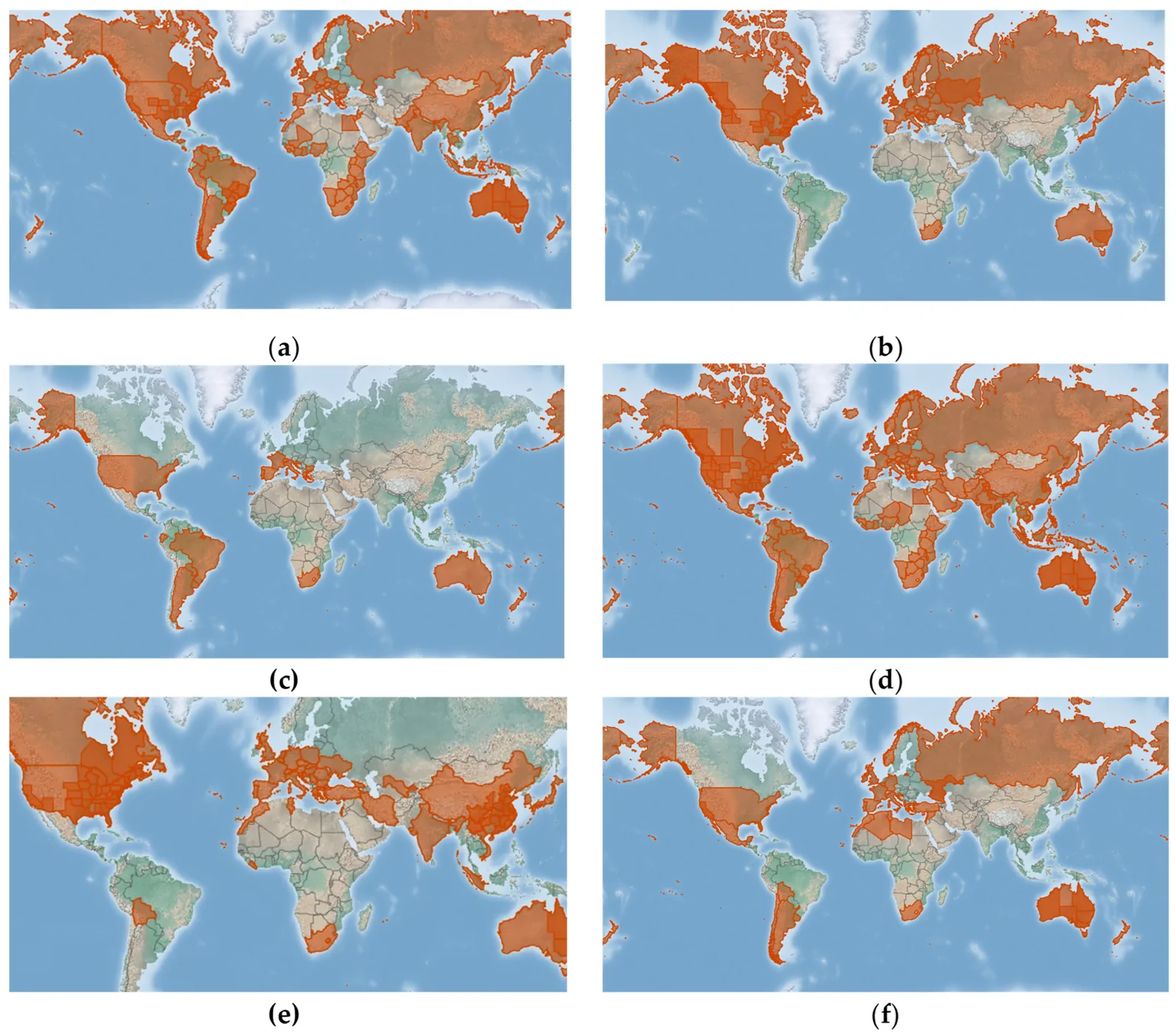
Distribution maps of (**a**) *Amaranthus hybrid,s* (**b**) *Reynoutria × bohemica,* (**c**) *Opuntia humifusa*, (**d**) *Oxalis corniculata*, (**e**) *Phytolacca americana*, and (**f**) *Carpobrotus edulis* [28].

*Amaranthus hybridus* (Family Amaranthaceae) is a native riverbank pioneer of eastern North America and parts of Mexico, Central America and northern South America [27]. Its distribution range has expanded to all continents [28,29] (Figure 1a). The plant occupies mainly ruderal and disturbed habitats but also penetrates natural habitats like the periphery of woods. It is close to another invasive alien relative *P. esculenta*, and is distinguished by its wide ovate leaves and eight-lobate fruits [9,30].

#### 2.1.2. *Reynoutria × bohemica*

*Reynoutria × bohemica* ([syn. *Fallopia × bohemica* (Chrtek & Chrtková) J.P.Bailey] [28], Family Polygonaceae) is the hybrid between *Reynoutria japonica* Houtt. (known as Japanese knotweed) and *Reynoutria sachalinensis* (F.Schmidt) Nakai and occurs at three different ploidy levels: 2n = 44; 66; and 88 [31,32,33,34,35,36]. This taxon spontaneously developed in Europe, outside the natural distribution of its parental species. Matkowsky and co-authors studied *R. japonica*, *R. sachalinensis*, and their hybrid *R. × bohemica* in Poland using SCoT and SRAP molecular markers, HPLC-MS metabolic profiling, and leaf morphology. They found that while morphology separates *R. sachalinensis*, it fails to distinguish hybrids from *R. japonica* [37,38]. The hybrid is listed among the top ten aggressive invasive plants. Although it originates from Europe, presumably as a result of spontaneous hybridization, it spreads fast all over the Northern Hemisphere and also in South Africa and Australia [28] (Figure 1b). The plant takes mainly ruderal habitats but also in natural habitats particularly near rivers and grasslands. It grows rapidly and densely and thus outcompetes the local plants [9].

#### 2.1.3. *Opuntia humifusa*

*Opuntia humifusa* [Syn. *O. vulgaris* Mill., *O. compressa* (Salisb.) MacBr. Family Cactaceae] is native to Central and South America [28,39]. It is particularly invasive in Southern Europe in recent decades, driven by climate change (Figure 1c). This species is also included in the worst ten invasive species. The cacti invasion is a serious problem for biodiversity in rocky and sandy habitats. It is a hazard for rare and endemic species [9]. Interestingly in the east it is known as the Korean cactus [40].

#### 2.1.4. *Oxalis corniculata*

*Oxalis corniculata* (Family Oxalidaceae) is native to Central and South America. It is invasive in all continents [28,41] (Figure 1d). The plant occupies mainly ruderal habitats but also grows in natural habitats particularly near rivers and grasslands. It grows rapidly and densely and thus competes with local plants [9].

#### 2.1.5. *Phytolacca americana*

*Phytolacca americana* (Family Phytolaccaceae) as the scientific name shows is native to the eastern parts of North America [28] (Figure 1e). The plant occupies mainly ruderal and disturbed habitats but also penetrates natural habitats like the periphery of woods. It is close to another invasive alien relative *P. esculenta*, and is distinguished by its wide ovate leaves and eight-lobate fruits [9,30,42].

#### 2.1.6. *Carpobrotus edulis*

*Carpobrotus edulis* (Family Aizoaceae) is native to South Africa. It inhabits coastal and inland slopes [43] (Figure 1f). Its ability to tolerate drought, salt, wind, and poor soils allows it to survive and establish easily in harsh coastal environments. *C. edulis* grows in dense, spreading mats that cover large areas and suppress native plants by competing for space, light, water, and nutrients. In invaded habitats, *C. edulis* can therefore form extensive populations and significantly alter native plant communities and ecosystem structure [43].

### 2.2. Ethnobotanical Data and Ecosystem Services

Ethnobotany provides valuable insights into the food uses of invasive alien plants and their potential contribution to ecosystem provisioning services. By documenting traditional and contemporary practices of harvesting and consuming these species, in their native ranges, ethnobotanical research can reveal underutilized food resources and their role in dietary diversity (Table 1).

#### 2.2.1. *Amaranthus hybridus*

*A. hybridus* (common pigweed, slim pigweed, slim amaranth) is a plant with a long history of consumption in Latin America, Africa, and parts of Asia [44]. The species is native to the Americas, where it has been cultivated and become a staple leafy vegetable and pseudocereal, especially in rural regions with limited resources [44,45]. The Indigenous peoples of the North American Southeast perceived wild *Amaranthus* spp. as a resource integrated into the traditional maize-bean-squash polyculture—often referred to as the ‘Fourth Sister’ [46,47]. It provides lysine, an essential amino acid that maize lacks, thereby completing the protein profile of a maize-based diet [48]. Among the Western Keres tribes (Acoma and Laguna), Slim amaranth was utilized as a primary leaf vegetable, young plants were boiled and eaten as greens or dried for winter use, while an infusion of the plant was administered as a gastrointestinal aid to treat stomach ailments [49,50,51]. In Cataract Canyon, the Havasupai utilized the leaves as a potherb, while seeds were ground and used as grain to make bread, soups and porridge or consumed as dumplings [51,52]. The Cherokee nation exploited the leaves of *A. hybridus* for their astringent properties to relieve profuse menstruation [53]. For the Cherokee and Iroquois tribes *A. hybridus* served as a ceremonial drug and a main ingredient of Green Corn Medicine (*Busk*) to achieve physical and spiritual purification [54].

In Mesoamerican cultures, especially Mexican, *A. hybridus,* Quintonil is managed as a wild edible green, or *quelite* [55,56]. Preparation of *quelite* (young branches or shoots, young leaves) dishes often involves frying them with oil, onions, and salt. Other popular preparations include steaming, roasting, or serving them in soups, sauces and gravies [56].

*A. hybridus* is a culturally significant crop among Andean communities with a long history of use, utilized as a staple leafy vegetable and pseudocereal [44,57]. In Ecuador the species is known as Sangorache or Ataco, where *A. hybridus* is a major ingredient in several traditional beverages [44,58]. In Loja province, Southern Ecuador, the inflorescence and young leaves provide the fuchsia color of the Horchata drink, a herbal mixture infusion consumed with meals, and served at home or sold at traditional markets. Horchata is believed to promote health, improve digestion and memory, and act as a hepatic anti-inflammatory and a diuretic agent [58]. The inflorescences of *A. hybridus* are a major ingredient of the ceremonial Ecuadorian *Colada Morada* beverage, consumed during the Day of the Dead celebrations. The red colored Amaranth in *Colada Morada* symbolizes blood, and thus life [44,58]. Local people of Loja province, Ecuador have consistently used inflorescences and young leaves of *A. hybridus* as an anti-flu, anti-inflammatory, diuretic, emmenagogue, tonic and vulnerary treatment [58]. Similar medicinal applications of *A. hybridus* were documented in the Zamora-Chinchipe province in Ecuador [59].

In Southern Africa *A. hybridus* is a primary constituent of the traditional leafy vegetable complex known as *marog* [60]. Within the Eastern Cape of South Africa, among the communities of the Nkonkobe Municipality *A. hybridus* (*imbuya)* serves as a critical nutritional resource [61]. The decoction from the leaves is traditionally administered for its astringent and antidiarrheal properties and is utilized as an anthelmintic agent [61]. In the Vhembe District of South Africa’s Limpopo Province *A. hybridus* (*vowa*, *thebe*, *gango*, *tshiubavhakololo*; *gewone misbredie*) is harvested as a fundamental leafy vegetable, providing essential nutrients in the local diet [62]. In Cameroon, *A. hybridus* (etang or country green) is used as leafy vegetable, while a maceration of its leaves and seeds is traditionally administered as a fertility stimulant [63,64,65].

The ethnobotanical utility of *A. hybridus* is documented in the Lesser Himalayas of Pakistan, where the species is harvested as a wild edible resource [66]. The leaves are prepared by boiling in water, while powdered seeds of the plant are traditionally administered orally to improve eyesight and treat visual impairments [66]. In the Kohistan Upper region of Pakistan, *A. hybridus* (gannar, ganaara, ganaar) is documented as a significant wild food resource [67]. Along with other food plants, it is gathered and frequently brought to the local markets. Fresh leaves are boiled in water or buttermilk, squeezed and fried with onion in butter [67].

#### 2.2.2. *Reynoutria × bohemica*

Since the highly aggressive invasive *R. × bohemica* is a relatively new taxon that spontaneously developed in Europe, outside the natural distribution of its parental species, there is no information about its traditional use. Therefore, it is worth summarizing the ethnobotanical information about its progenitors. Japanese knotweed (*Reynoutria japonica* Houtt.) is eaten raw with salt and the stems of the female plants are preferred or cooked instead of bamboo sprouts [68,69]. In Japan children forage the sour-tasting leaves, shoots and stems of the plant and eat them raw [68]. In Japan, Kochi Prefecture, Japanese knotweed (itadori) is a familiar food, and it is used in a variety of dishes, such as stir-frying, simmered dishes, vinegared dishes, and chirashi-zushi [70]. It is gathered from the wild and sold at supermarkets or in direct sales stands, and roadside stations in Kochi Prefecture. The sprouts are pickled in salt or frozen in order to be served outside of the harvesting season [70]. In Japan, *F. japonica* is also valued as a melliferous plant. Its late summer blossom serves as a vital source of nectar for local beekeeping [71]. The produced monofloral honey, known as “Bamboo honey” is dark, with an intense flavor and prominent antioxidant capacity [72]. The honey was produced first in Japan, where the plant is native and later, in newly invaded territories and has maintained the common use of the plant in Europe and America [73].

In the Shennongjia region of central China fresh shoots of Japanese knotweed have been consumed for centuries as a traditional vegetable, known as Huangyagan or suantonggan). They are typically boiled before eating [74]. Local residents believe that the plant has biological effects, such as antibacterial, anti-aging, and antioxidant properties [74]. In some regions of Southern and Central China, the root extract is used as a natural pigment to dye rice, a practice facilitated by the high concentration of anthraquinones [75]. In India and Southeast Asia dried leaves of Japanese knotweed are used as a tobacco substitute [76].

In the territories of its origin Japanese knotweed has been prized as a medicinal plant for centuries. The dried root is a well-known traditional Chinese medicine (*Huzhang*) officially listed in the Chinese Pharmacopeia [75,77]. Currently, in China, the herb is used in the form of powders, decoctions or infusions as a treatment for inflammatory diseases (including hepatitis, suppurative dermatitis) as well as favus, jaundice, skin burns, scald and hyperlipemia [75]. It is widely cultivated as an essential medicinal plant in many provinces of China (including Anhui, Fujian, Gansu, Guangdong, and Guangxi), in contrast with its status as an invasive plant in Europe and America [78].

#### 2.2.3. *Opuntia humifusa*

*Opuntia humifusa* (Eastern Prickly Pear, Devil’s-tongue) served as a critical seasonal and survival food source for Indigenous peoples across Eastern and Central North America. The Dakota and Pawnee tribes traditionally harvested young stems (cladodes); once cleared of spines, these were peeled, roasted, and consumed during periods of food scarcity [79,80]. The ripe fruits (tunas) were eaten raw, boiled into syrup, or dried for winter storage [79,80]. Peeled stems were applied as a poultice on wounds. The Lakota and Southern Appalachian people used Eastern Prickly Pear as a food source in a similar manner, while cut stems were applied locally as a rattlesnake bite remedy in the Lacota community [80,81,82]. The Nanticoke Indians named *O. humifusa* “wart-plant” and rubbed the juice of the fruits on warts [80,83]. Mucilaginous stem juice was used to fix colors painted on hides or receptacles [79,80]. *O. humifusa* played a central role in the Dakota summer “cactus game,” which served as a test of a boy’s bravery and stoicism [79,80]. The Rappahannock tribe utilized *O. humifusa* as a spiritual guardian, believed to repel negative spirits [84].

*O. humifusa* (*Cheon-nyeon-cho*) is valued as a traditional biological resource in Korea. The fruit extracts of *O. humifusa*, are used to treat pulmonary inflammation, chronic cough and bronchial asthma [85]. They are harvested when deep purple and typically macerated with sugar to form Cheong, a cactus syrup [86]. The fresh mucilaginous fiber from the cladodes is consumed to promote bowel movements and alleviate chronic constipation [87]. The Compendium of Materia Medica, *Boncho Gangmok*, prescribes the fresh juice of stems for the regulation of blood glucose levels [88].

In contemporary Korean gastronomy, *O. humifusa,* the ‘thousand-year plant’, has transitioned from a folk remedy into a prized functional food. The deep purple fruits are processed into a fine powder used as a natural pigment for *Sulgi-tteok*, traditional ceremonial rice cakes [86].

The production of cactus syrup, “Cheonnyuncho-cheong”, represents the traditional Korean food preservation technology that remains widely utilized in the modern daily diet. The fruits of *O. humifisa* are layered with sugar and aged for three months [89]. The process of osmotic maceration results in a dense syrup, rich in betalains and Vitamin C, which is consumed as a health tonic to support immunity and promote energy, or is utilized as an ingredient in meat marinades, salad dressings, and various traditional desserts [89].

#### 2.2.4. *Oxalis corniculata*

*Oxalis corniculata* commonly known as Indian sorrel, wood sorrel or chengeri tenga is a garden weed found throughout India [90]. Its leaves serve as the major source of food for the tribal populations during severe famine and extreme scarcity. The leaves are eaten raw, by adding them to salads or cooked with other vegetables to give them a sour flavor. They are also used to prepare chutney abd pickles by the Sahariya tribe in Central India [90]. Leaf decoction is used for fever and dysentery in Madhya Pradesh, India [91].

The leaves of the plant are considered cooling, antiscorbutic, and astringent and are useful in treating fever, biliousness, dysentery and prolapse of the rectum [92]. The wood sorrel has been used in treating liver and digestive issues as mentioned in early Ayurvedic medical writings [93]. Leaf extract is used to treat burns, bug bites and various skin outbreaks, aids in the treatment of boils, warts, corns, and inflammation. Infants with hookworms are treated with this herb for its anthelminthic properties. Jaundice is treated with leaf juice, which is also quite efficient in managing diabetes. Leaf infusion is used to treat fevers of mild and high grades and helps relieve corneal opacity and eye pain [93]. Leaf paste is physically rubbed onto the forehead to cure headaches, and the leaf extract is used to alleviate insomnia via massage [91,93]. The herb juice of *O. corniculata* (*Sengri meghi*) is used as an eye drop in conjunctivitis by Boro tribal people of Assam, northeastern India [94]. In Balasore district of Odisha, India, Santal tribal people use leaves for treating diarrhea in children [95]. In Nepal the entire plant is used as medicine, and sensitive teeth can be treated with this shrub [93]. In rural areas of Azad Jammu and Kashmir, Pakistan the juice and decoction of fresh leaves and roots of *O. corniculata* (*Khatkoira*) was documented for treating diabetes, being one of the most frequently reported species [96].

In Oman, the plant is considered edible with anti-diabetic properties [97]. In Tanzania, where it is known as the creeping wood sorrel, extracts of the plant are used traditionally as medicine and as food [98]. It is used to treat wounds, as a tonic for various gastrointestinal ailments and diarrhea, especially in infants, to stop stomach upset and nausea especially for pregnant mothers to stimulate their appetite, for heartburn, flu, tonsillitis and coughs, and for treating children’s first milk tooth irritation and aching [98]. In the rural area of Ndop, Republic of Cameroon, whole plant maceration of Creeping wood sorrel (*Seineh*), mixed with *Sida acuta* leaves is taken orally to treat gonorrhea [99].

#### 2.2.5. *Phytolacca americana*

*Phytolacca americana* (Pokeweed, poke sallet, polk salad) is a traditional, foraged food for native North American tribes. The young shoots, leaves and stems were cut, parboiled, rinsed and cooked, mixed with other greens and eggs, while peeled stalks, cut lengthwise, are dipped in egg and fried by the Cherokee tribe [100]. Pokeweed meals are believed to clean the blood and can serve as spring tonic for both treating and preventing illness [101]. The leaves are gathered in the early spring and can also be dried for future use while crushed berries are utilized as food colorant and as a beverage mixed with strained sour grapes and sugar [100]. Fruit juice of Pokeweed was used by Pawnee people as paint for horses and various articles of adornment [79]. Native Americans used Pokeweed as a poultice for skin diseases, sores, ulcers, and tumors. The plant roots are cut and strung around the baby’s neck for cutting teeth [102]. Traditional healers used plant roots, boiled with sugar as a cure for asthma and colds, as a sedative, and as a treatment for gout and rheumatism [102]. Rappahannock people used the steam from decoction of roots as a hemorrhoid remedy, while in Southern Appalachian folk medicine berry juice is used for the same purpose [84,101].

*P. americana* was introduced to Europe in the 17th century due to its ornamental and tinctorial properties. It was quickly commercialized as a ‘correcting’ agent for the visual quality of red wine [103]. The practice was legally prohibited to prevent accidental intoxication due to the high triterpenoid saponin content of the plant [102,104].

In the newly invaded territories, the pokeweed is used as a novel, “unusual” plant for “sarma” preparation in the Republic of North Macedonia [105], and in SW Bulgaria [24], representing the dynamic nature of folk cuisines.

#### 2.2.6. *Carpobrotus edulis*

In South Africa, the fruits of *Carpobrotus edulis* (Sour fig, Suurvy, Ghaukum, Ghoenavy) are traditionally consumed fresh or dried [106]. The fruits have served as a vital food source for the nomadic Khoi and San groups, while the Zulu community perceives them as a supplementary food [60]. Sour figs play a key role in Cape Malay culinary tradition, which includes a jam served as a delicacy at weddings and Eid (Sour figs konfyt, *Suurvye konfyt*), and the preparation of aromatic pickles (*atjars*) [107,108]. The dried fruits of the sour fig are still sold at Cape vegetable markets, representing a means of income for local communities who harvest them from the wild [60,107].

Medicinally, *C. edulis* has been utilized for centuries by the Khoisan, Zulu, and Xhosa communities in South Africa. Leaf juice is used as gargle for diphtheria, sore throat, mouth infections, vitiligo and excessive salivation and is swallowed for dysentery and diarrhea [28,109,110,111]. A local application of leaf juice over burns and scalds is commonly used today [109]. Khoisan women consumed fruit infusions during pregnancy for an easy birth and applied leaf juice to newborns to make them strong and nimble [112,113]. South African farmers have utilized the tincture or decoction of the whole plant as a sedative [114]. The plant is also used as a remedy for several infectious diseases, including tuberculosis [112,113]. Traditional healers in Nkonkobe Municipality recommend roots and leaf decoctions of *C. edulis* to manage HIV/AIDS symptoms, particularly to remove shingles from HIV patients and boost the immune system [115]. Other diseases treated are diabetes mellitus, high blood pressure, intestinal worms and constipation [112]. In the Eastern Cape the plant was also used to fight fatigue, vaginal thrush, toothache, and earache, and for spiritual purposes [106,116].

In Europe, *C. edulis* was introduced for landscaping and to stabilize and fix coastal sand dunes, and for landscaping [117]. The fresh juice has been used to treat water retention and painful urination, and to soothe lung inflammation, with contemporary research validating its potent antibacterial and antioxidant properties [118,119,120]. In coastal areas, *C. edulis* is used as a soothing remedy for bluebottle stings and for treating wounds and burns [120]. Furthermore, its high water content (90–95%) has led to its use as a natural fire barrier (fire retardant plant) in wildfire-prone areas, such as California, where it is planted as a groundcover around houses [121].

**Table 1:** Ethnobotanical data of the consumption traditions and practices.

Plant Species	In the Range of Origin	Worldwide
*Amaranthus hybridus*	**North America** **Native American tribes** (*Slim amaranth*) food (leaves; seeds), medicine (gastrointestinal aid; relieve profuse menstruation), ritual use [49,50,51,53,54] **Mexico** (*Quintonil blanco*, *bledo*) food (leaves, stems, seeds) [55,56] **South America** **Ecuador** (*Sanquorache*, *ataco*, *bledo*) food (leaves, seeds), beverage, ritual use, medicine (anti-inflammatory, diuretic, emmenagogue, tonic, vulnerary; cold, headache, stomach pain) [44,45,57,58,59]	**Africa** **South Africa** food (leaves) [60] **South Africa**, Eastern Cape (*Imbuya*) food (leaves), medicine (astringent, anthelmintic, antidiarrheal) [61] **South Africa**, Limpopo Province (*Vowa*, *gewone misbredie*) food (leaves) [62] **Cameroon** (*Etang*, *Country green*) food (leaves), medicine (fertility stimulant) [63,64,65] **Asia** **Pakistan, Lesser Himalayas** food (leaves), medicine [66] **Pakistan, Kohistan** (*Gannar*, *ganaara*, *ganaar*) food (leaves) [67]
*Reynoutria × bohemica*	**Asia, Japan** (*Itadori*) food, children snack (stems, shoots; leaves), melliferous plant medicine (anti-inflammatory aid) [68,69,71,72,73] **China** (*Huangyagan*, *suantonggan*) food, food dye (stems; root extract) medicine (anti-inflammatory aid) [75,78] **India** tobacco substitute (leaves) [76]	**Europe** melliferous plant (nectar producing) [72,73]
*Opuntia humifusa*	**North America** **Native American tribes** (*Eastern Prickly Pear*, *Devil’s-tongue*) food, famine food (stems, fruits) medicine (stems, locally on wounds, snake bites, fruits: for warts); color mordant (stem juice) ritual use [79,80,81,82,83]	**Asia, South Korea** (*Cheon-nyeon-cho*) food (fruits, stems), functional food; ceremonial food (*Sulgi-tteok* rice cakes) medicine (fruit extracts: pulmonary inflammation, chronic cough, bronchial asthma; stem juice: constipation, glucose levels regulation) [85,87,122]
*Oxalis corniculata*	**Asia** **India** (*Indian Sorrel*, *Chengeri tenga*) food (leaves) medicine (leave decoction: fever, dysentery; leaf extract: burns, bug bites; for headaches and insomnia, relieve eye pain; anthelminthic for children, for jaundice; diarrhea in children [90,91,94,95] **Nepal** medicine (sensitive teeth treatment) [93] **Pakistan** (*Khatkoira*) medicine (leaf juice, decoction of fresh leaves and roots, for treating diabetes) [96] **Oman** food (leaves) medicine (antidiabetic agent) [97]	**Africa** **Tanzania** (*Creeping wood sorrel*) food (leaves) medicine (diarrhea, anti- nausea in pregnant mothers, for heart burn, flu, tonsillitis and cough) [98] **Cameroon** (*Seineh*) medicine (whole plant maceration with *Sida acuta* leaves to treat gonorrhea) [99]
*Phytolacca americana*	**North America** **Native American tribes** (*Pokeweed*, *poke sallet*) food (leaves, shoots, stems, stalks), beverage, food colorant, paint (fruits), medicine (leaves: skin diseases, sores, ulcers; root decoction: gout, rheumatism, as sedative; steam from decoction, fruit juice: hemorrhoids) [79,84,100,101,102]	**Europe** ornamental, food additive (fruit juice in wines) [103] **Republic of North Macedonia** food (leaves, for sarma preparation) [105]
*Carpobrotus edulis*	**South Africa** (*Sour fig*, *Suurvy*, *Ghoenavy*) food (fruit, leaves) medicine (leaf juice, diphtheria, sore throat, mouth infections; dysentery, diarrhea; infectious diseases, tuberculosis, to manage HIV/AIDS symptoms; diabetes mellitus, high blood pressure, intestinal worms, constipation; sedative spiritual use [106,109,111,112,113,114,116]	**Europe** landscaping (stabilizing coastal dunes) medicine (leaf juice: painful urination, lung inflammation; bottle stings bites, treating wounds, burns) [117,118,119,120] **North America** **USA, California** fire retardant plant [121]

### 2.3. Bioactive Compounds, Pharmacological Effects and Toxicity

The phytochemical and pharmacological investigation of the selected invasive alien plants has revealed a diverse range of bioactive and biological compounds. Table 2 summarizes the plant parts investigated, the reported bioactive compounds and pharmacological effects, and the available evidence regarding toxicity, while distinguishing experimental findings from established traditional food uses and highlighting the current limitations of the evidence.

#### 2.3.1. *Amaranthus hybridus*

Phytochemical investigations on *A. hybridus* indicate that the species contains a range of secondary metabolites, although the number of studies specifically addressing isolated compounds remains limited. Preliminary analyses of aerial parts have demonstrated the presence of phenolic compounds, flavonoids, tannins, saponins, terpenoids and steroids, with rutin detected by TLC in hydroacetonic extracts and the highest total phenolic content observed in the same fraction [123]. Betalains have also been reported, supporting the antioxidant potential of the species [123].

**Figure 2:**
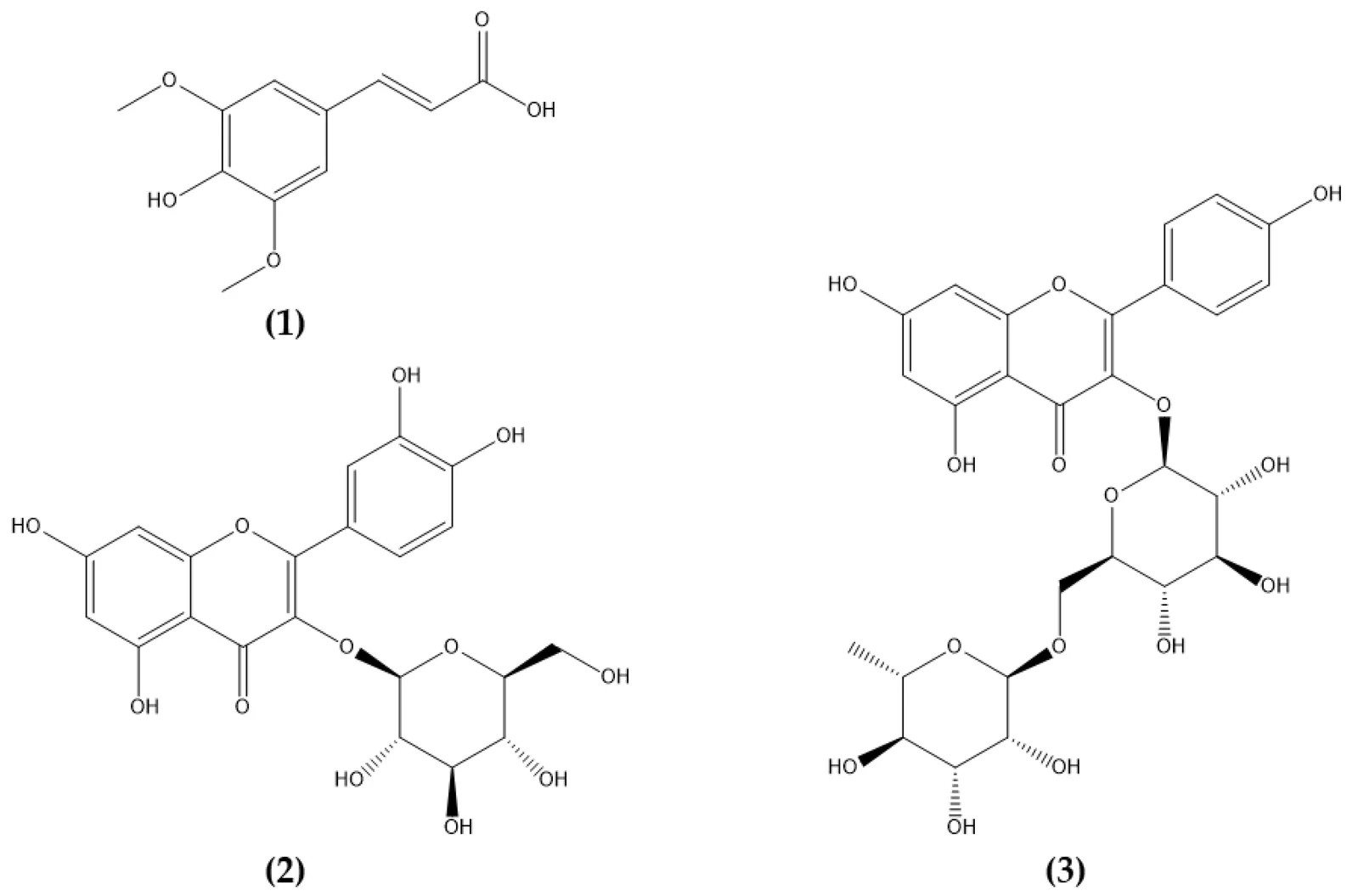
Notable phytochemicals in *A. hybridus*. Sinapic acid (**1**), Isoquercetin (**2**), and Kaempferol-3-*O*-*β*-rutinoside (**3**).

Another study examined the chemical composition of the leaves of the species using UHPLC-PDA and revealed the presence of several hydroxybenzoic acids (gallic acid, 3,4-hydroxybenzoic acid, vanillic acid, etc.) and hydroxycinnamic acids (caffeic, *p*-coumaric, furiluc, sinapic (Figure 2(**1**)) and cinnamic acid). Additionally, rutin, isoquercetin (Figure 2(**2**)), kaempferol-3-*O*-*β*-rutinoside (Figure 2(**3**)), quercetin and kaempferol were also identified [124]. Polysaccharides from the aerial parts of the plant were also isolated. The monosaccharide composition analysis was carried out via GC-MS and revealed mannose, rhamnose and glucose as the backbone of the substance [125]. Several general tests revealed the presence of alkaloids in the species [126].

Pharmacological studies, specifically on *A. hybridus* remain limited but suggest potential antidiabetic activity. A methanolic extract of the species has been shown to stimulate glucose uptake in L6 myoblasts and C3A hepatocytes (15.95% at 31.25 µg/mL), although with lower efficacy compared to insulin, indicating moderate bioactivity [127]. In contrast, strong α-glucosidase inhibitory activity has been reported for extracts of *Amaranthus* species; however, these findings derive from multi-species studies and cannot be exclusively attributed to *A. hybridus* [128].

The antioxidant activity of polysaccharides isolated from *Amaranthus hybridus* L. has been demonstrated in vitro, showing significant radical scavenging activity against DPPH, hydroxyl, and superoxide radicals, with effects comparable to vitamin C [125]. In vivo studies further suggest that dietary supplementation with *A. hybridus* may mitigate heavy metal-induced toxicity in animal models, reducing histopathological damage in the liver, kidney, and intestinal tissues following exposure to iron, lead, and cadmium [129].

An acute toxicity assessment using brine shrimp lethality assays indicates the low toxicity of the methanolic extracts of this species [126]. However, isolated evidence from a veterinary case report has suggested potential toxicity in cattle associated with *A. hybridus* consumption, although causality remains uncertain and may depend on environmental and feeding conditions [130]. Toxicological data suggest low acute toxicity for crude extracts of *A. hybridus*, as indicated by brine shrimp lethality assays [126]. Nevertheless, comprehensive toxicological characterization remains limited.

#### 2.3.2. *Reynoutria × bohemica*

Phytochemical studies on *R. × bohemica* have revealed that it contains a chemically diverse range of secondary metabolites. Comparative HPLC-DAD profiling of methanolic fine-root extracts showed complex phenolic fingerprints, with compounds assigned to the flavone, flavonol, hydroxycinnamic acid, anthraquinone, and stilbene classes [131]. Seasonal variation in tannin and anthraquinone content was also investigated in leaf and rhizome samples collected before, during, and after flowering. In *R. × bohemica*, tannin levels were generally higher in the leaves than in the rhizomes, whereas anthraquinones were determined in the rhizomes. Before flowering, rhizome anthraquinone content was highest in *R. japonica*, followed by *R. × bohemica and R. sachalinensis* [132].

**Figure 3:**
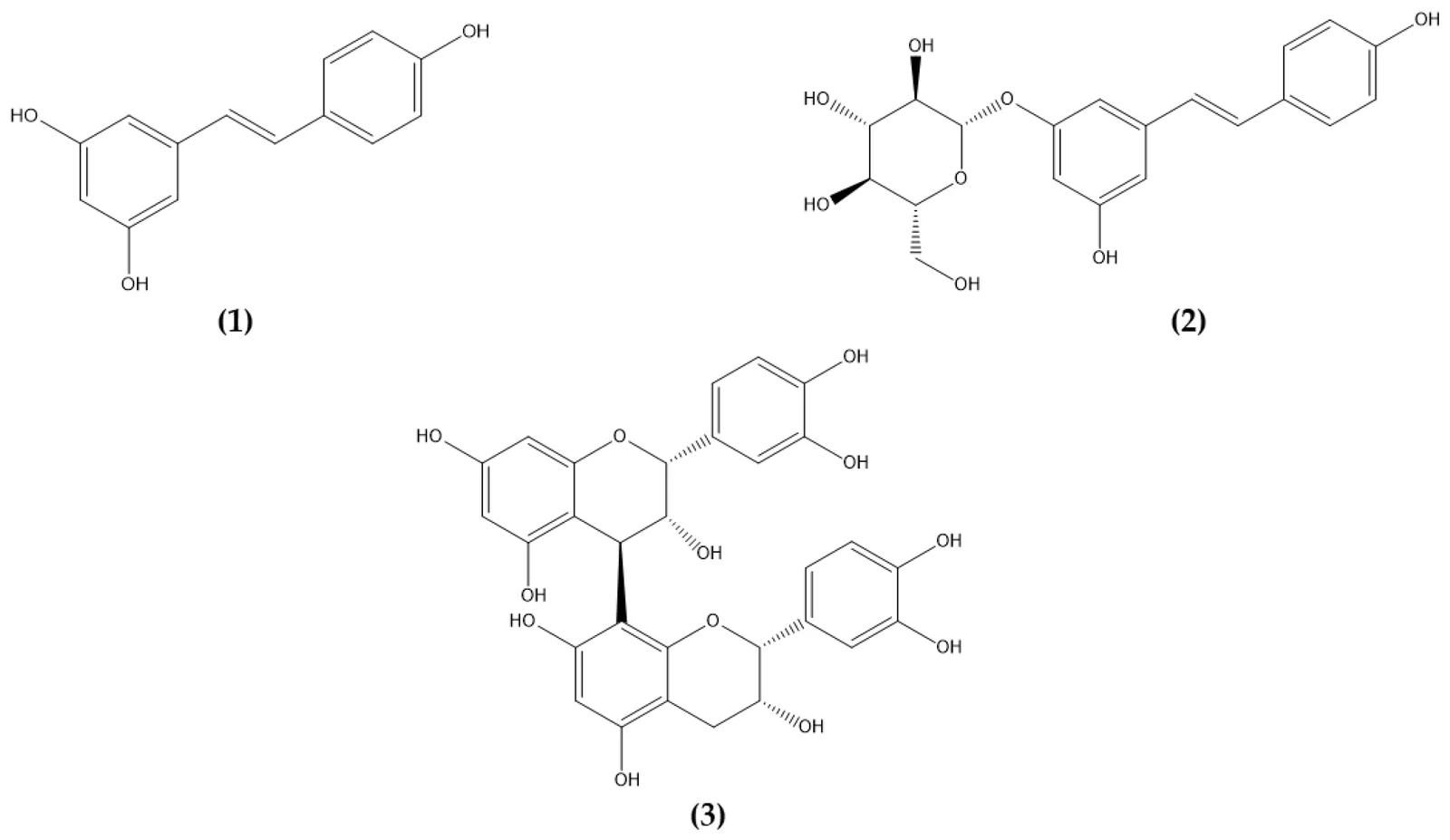
Notable phytochemicals in *R. × bohemica*. (**1**) Resveratrol, (**2**) Polydatin, (**3**) Procyanidin B2.

The chemical characterization of *R. × bohemica* rhizomes was performed by quantitative ^1^H NMR analysis of ethanolic extracts, resulting in the identification and quantification of three secondary metabolites: resveratrol (Figure 3(**1**)), (−)-epicatechin, and emodin [133]. The study reported comparable epicatechin and emodin levels in *R. japonica* and *R. × bohemica*, whereas the latter contained approximately 24% more resveratrol. Complementary analyses based on high-performance thin-layer chromatography coupled with densitometry, image analysis, and tandem mass spectrometry confirmed the presence of (+)-catechin, (−)-epicatechin, (−)-epicatechin gallate, and procyanidin B2 (Figure 3(**3**)) in the rhizomes of *R. × bohemica*. These compounds were reported for the first time in the rhizomes of this hybrid species [134]. Polydatin, the 3-*O*-*β*-D-glucoside of resveratrol (Figure 3(**2**)), was also identified in rhizome extracts by HPLC-DAD [135].

The mature green leaves of *R. × bohemica* may also represent a valuable source of carotenoids. Their total carotenoid content was reported as 260 mg of lutein equivalents per 100 g of dry weight [136]. This value applies specifically to mature green leaves, whereas substantially lower concentrations were found in senescing plant material. *Reynoutria × bohemica* exhibits an intermediate phytochemical profile between its parent species, *R. japonica* and *R. sachalinensis*, characterized by the presence of stilbenes, anthraquinones, flavan-3-ols, and phenylpropanoid esters [137].

Ethanolic extracts obtained from the rhizomes and flowers of *R. × bohemica* were evaluated for several in vitro biological activities in a comparative study that also included *R. japonica*. Cytotoxicity was assessed in PaTu, HeLa, and HepG2 cancer cell lines, with HEK293T cells included as a non-tumor control. Neither the rhizome nor the flower extract showed cancer-selective cytotoxicity. Their effects were instead consistent with a non-selective toxic mode of action, although the flower extract was generally less cytotoxic than the corresponding rhizome extract.

*R. × bohemica* rhizome extracts, namely the ethyl acetate and diethyl ether fractions, demonstrates potent in vitro antiglycoxidative activity, comparable to or exceeding aminoguanidine, suggesting potential for preventing advanced glycation and oxidative protein damage associated with diabetes mellitus [138]. All the investigated extracts inhibited α-amylase activity in a concentration-dependent manner, with rhizome extracts generally displaying greater activity than flower extracts. Nevertheless, the strongest inhibition in the overall comparison was observed for the *R. japonica* rhizome extract; therefore, the highest activity cannot be attributed specifically to *R. × bohemica*. In the cellular antioxidant activity assay, performed at an extract concentration of 1 µg/mL, the *R. × bohemica* flower extract exhibited the highest cellular antioxidant capacity among the samples tested [135].

#### 2.3.3. *Opuntia humifusa*

**Figure 4:**
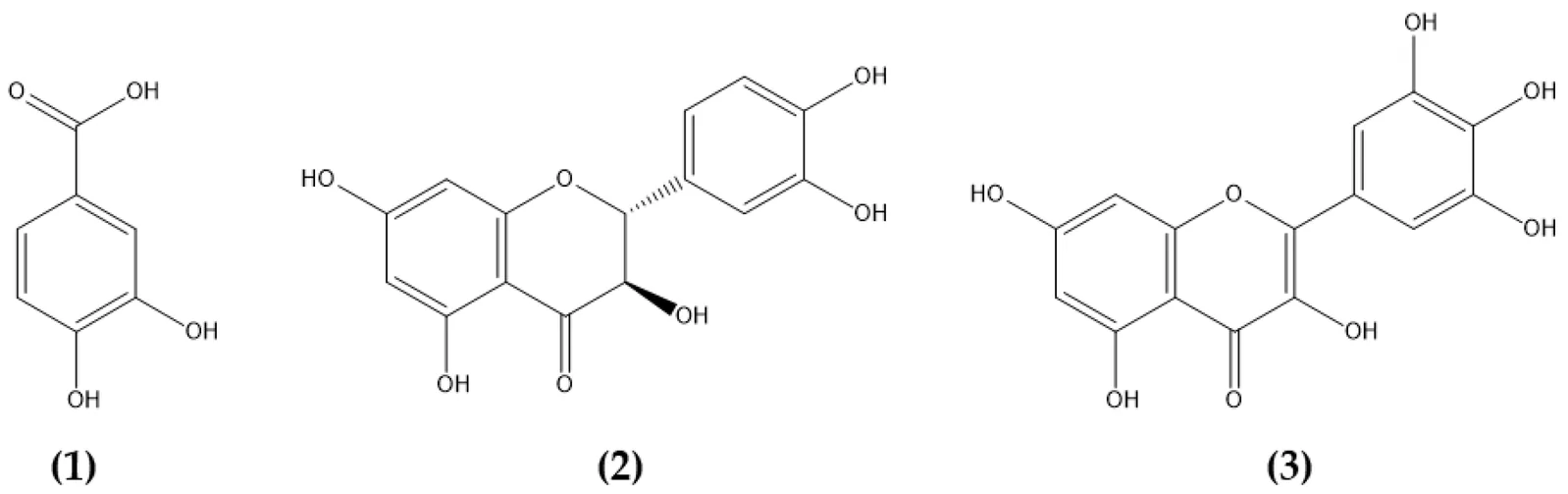
Notable phytochemicals in *O. humifusa*. Protocatechuic acid (**1**), Taxifolin (**2**), and Myricetin (**3**).

The phytochemical analysis of the *O. humifusa* fruit was performed on an 80% aqueous ethanolic extract that was subsequently partitioned using solvents of increasing polarity. Among the resulting fractions, the ethyl acetate fraction showed the highest total phenolic and flavonoid contents and was therefore further characterized by HPLC-DAD. Sixteen phenolic compounds were detected, including eleven phenolic acids and five flavonoids. Ferulic acid was the most abundant phenolic acid, with a concentration of 3.55 mg/g of fraction, followed by protocatechuic acid (Figure 4(**1**)) and *p*-coumaric acid at 1.79 and 1.53 mg/g, respectively. Taxifolin (Figure 4(**2**)) and myricetin (Figure 4(**3**))were the predominant flavonoids, occurring at concentrations of 5.83 and 3.06 mg/g, respectively [40].

The presence of taxifolin in *O. humifusa* was independently confirmed through activity-guided isolation from the ethyl acetate fraction of a 70% ethanolic extract. The purified compound was structurally identified as taxifolin by electron-impact mass spectrometry and ^1^H and ^13^C NMR spectroscopy. The isolated compound also exhibited marked radical-scavenging activity, comparable to that of established antioxidant reference compounds under the same experimental conditions [122].

Extracts prepared from different parts of *O. humifusa* have been investigated in several preclinical models. In U87MG human glioblastoma cells, the effects of hexane- and ethylacetate-soluble extracts and a water-partitioned fraction were assessed using the MTT assay. In contrast, changes in cell-cycle distribution and cell death were examined by flow cytometry. Treatment with the hexane-soluble extracts and the water-partitioned fraction reduced cell viability in a concentration-dependent manner. In particular, the water fraction induced cell-cycle arrest in the G1 phase, promoted predominantly non-apoptotic cell death, and increased intracellular reactive oxygen species production. These results exhibit an in vitro antiproliferative effect against U87MG cells, although they do not establish therapeutic efficacy against glioblastoma [139].

The antioxidant and anti-inflammatory properties of *O. humifusa* leaves were also investigated using a methanolic extract and its hexane, chloroform, ethyl acetate, butanol, and water fractions. Except for the aqueous fraction, the tested samples displayed radical-scavenging activity. The ethyl acetate fraction showed the strongest activity, with IC_50_ values of 3.6 µg/mL in the xanthine oxidase assay and 48.2 µg/mL in the DPPH radical-scavenging assay. Activity-guided fractionation further identified quercetin as one of the compounds contributing to the antioxidant effect of this fraction [140].

In LPS-stimulated RAW264.7 murine macrophages, the chloroform and ethyl acetate fractions significantly reduced nitric oxide production and suppressed the expression of inducible nitric oxide synthase and interleukin-6. The ethyl acetate fraction additionally reduced interleukin-1β expression. These data suggest that the two fractions can modulate selected inflammatory mediators in vitro, although their pharmacological relevance requires confirmation in appropriate in vivo and clinical models [140].

A systematic review and meta-analysis reported the available preclinical evidence concerning the biological properties of *O. humifusa*, including effects on cancer- and diabetes-associated experimental endpoints. The studies reported reduced proliferation in several cancer cell models and improvements in glucose and lipid-related parameters in diabetic animals. However, most of the available evidence was derived from in vitro experiments or animal models, and the authors noted considerable heterogeneity and limited statistical strength. Consequently, the available findings should be regarded as preliminary evidence of potential biological activity [89].

The short-term oral toxicity of an *O. humifusa* extract was evaluated in male and female Sprague–Dawley rats following a single administration and a subsequent repeated administration for 14 days. Animals received doses of 500, 1000, or 2000 mg/kg body weight/day, and the single-dose assessment included administration of 2000 mg/kg body weight. No mortality, treatment-related clinical signs, abnormal gross lesions, or relevant changes in body weight, food and water consumption, relative organ weights, hematological parameters, serum biochemical indices, or histopathological findings were observed. Under the conditions of this short-term study, the no-observed-adverse-effect level was greater than 2000 mg/kg body weight/day in both sexes. Nevertheless, these conclusions cannot be directly extrapolated to chronic exposure or to safety in humans [141].

#### 2.3.4. *Oxalis corniculata*

**Figure 5:**
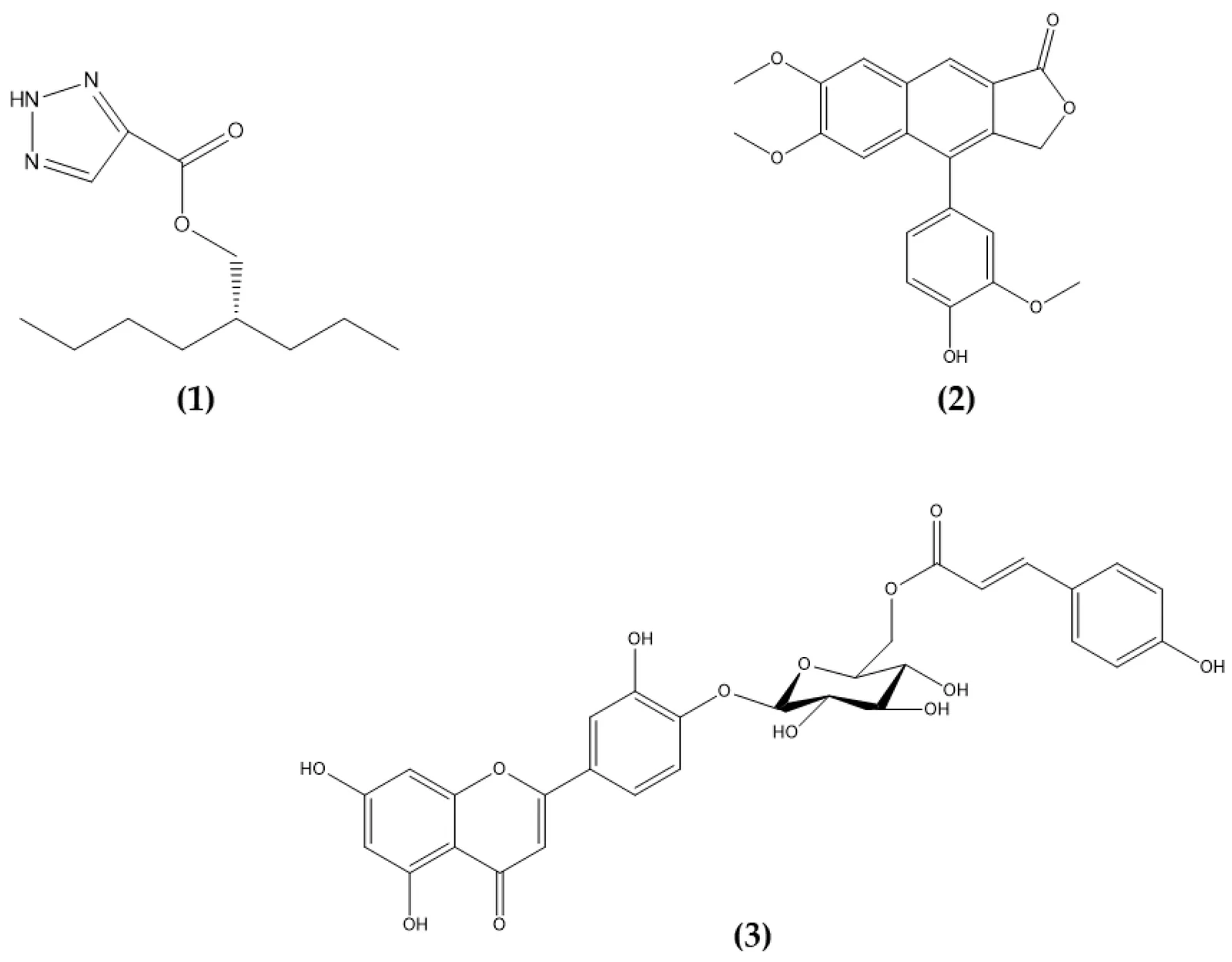
Notable phytochemicals in *O. corniculata*. Aspergillus triazolate A (**1**), Corniculin (**2**), and Corniculatin A (**3**).

Numerous phytochemicals have been identified in *O. corniculata*, particularly in its aerial parts and leaves. Recent phytochemical surveys indicate that the species is characterized by a complex metabolite profile dominated by flavonoids, organic acids, terpenoids, and alkaloids [142]. Among the flavonoids, flavone and flavonol glycosides appear to be especially abundant. In this context, corniculatin A (Figure 5(**3**)), identified as luteolin-6″-(*E*-p-hydroxycinnamoyl)-4′-*O*-*β*-D-glucopyranoside, was first isolated from the aerial parts of the plant [143]. A structurally unusual alkaloid, termed aspergillus triazolate A (Figure 5(**1**)), was also reported for the first time from *O. corniculata* [144]. In addition, coumarins, polysaccharides, and anthraquinones have also been described among the phytochemical constituents of the species [142]. Further expanding the known chemical diversity of the plant, the structure of corniculin (Figure 5(**2**)), a new arylnaphthalide lignan isolated from a 75% ethanolic extract of *O. corniculata*, was elucidated by mass spectrometry together with 1D and 2D NMR techniques [145].

Preclinical studies have shown that *O. corniculata* extracts and several of their secondary metabolites display a broad spectrum of biological activities, including anti-inflammatory, antimicrobial, hepatoprotective, neuroprotective, and antiproliferative effects. A hot-water extract of the plant was reported to exert anti-inflammatory activity in vitro by suppressing iNOS and COX-2 and by reducing TNF-α and IL-6 levels in a dose-dependent manner [146]. In addition, ethanolic and ethyl acetate extracts of the species have shown in vitro antiproliferative or cytotoxic activity against MCF-7 breast cancer cells and HepG2 hepatocellular carcinoma cells [147,148]. The newly described alkaloid aspergillus triazolate A also exhibited marked α-glucosidase inhibitory activity, with an IC_50_ value of 66.84 ± 1.50 µM, and was further reported to reduce postprandial blood glucose levels in vivo at a dose of 20 mg/kg [144].

The safety profile of *O. corniculata* has also been investigated in vivo. Oral administration of plant extracts at doses of up to 2000 mg/kg did not produce evident behavioral abnormalities or overt toxic effects in Swiss albino mice, suggesting a relatively low acute toxicity under the tested experimental conditions [149]. Nevertheless, the reported presence of calcium oxalate in the plant should be taken into consideration [150]. For this reason, caution may be warranted in individuals predisposed to nephrolithiasis, particularly in the case of prolonged or excessive consumption [13].

#### 2.3.5. *Phytolacca americana*

**Figure 6:**
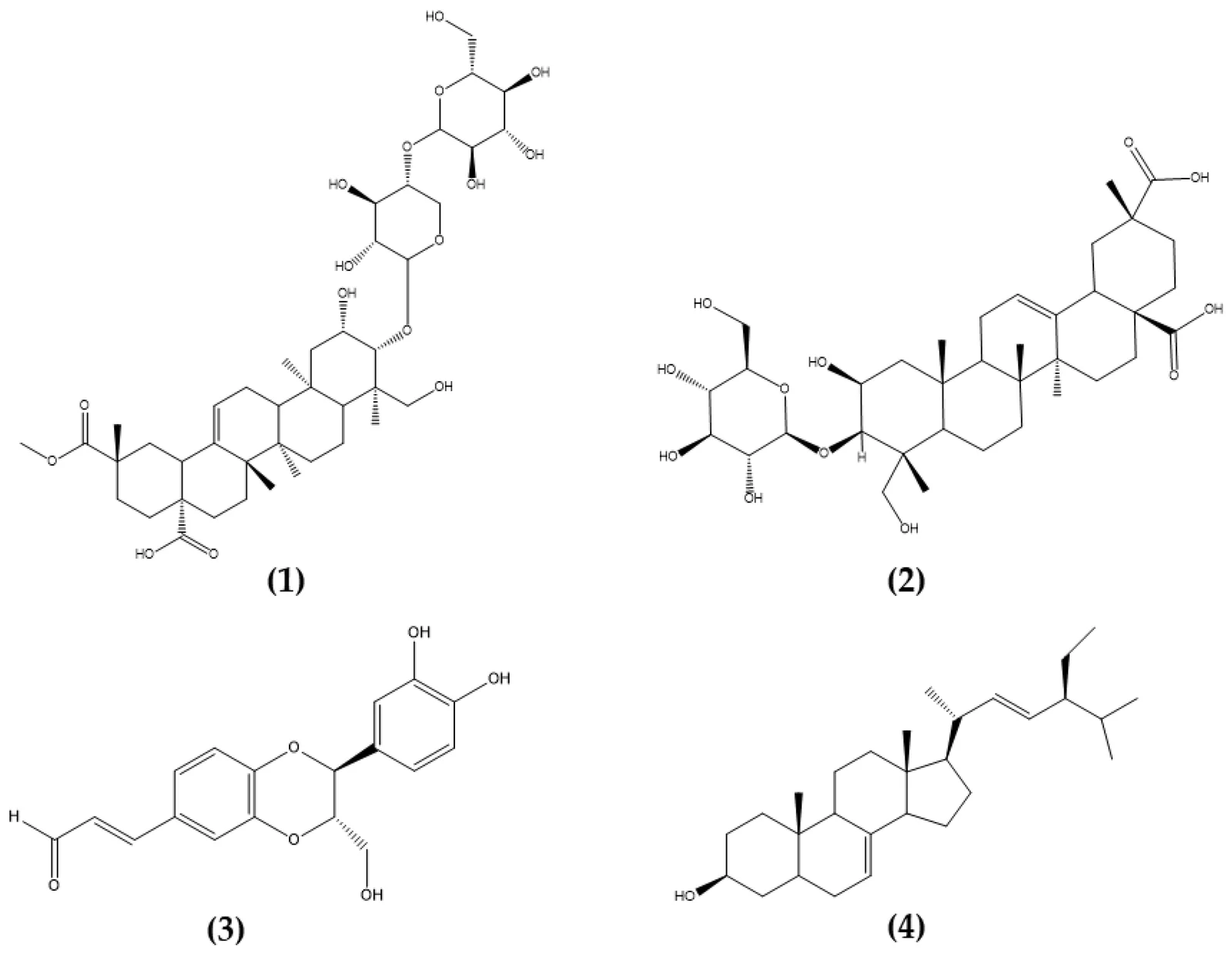
Notable phytochemicals in *P. americana*. Esculentoside A (**1**), Esculentoside P (**2**), Americanin A (**3**), and *α*-spinasterol (**4**).

*P. americana* is characterized by a chemically diverse phytochemical profile that includes triterpenoid saponins, lignans and neolignans, flavonoids, phytosterols, and several additional classes of secondary metabolites [151]. Among its most characteristic constituents are the esculentosides (Figure 6), a group of oleanane-derived triterpenoid saponins reported in several species of the genus *Phytolacca*. Although these compounds represent an important component of the genus, they should not be regarded as the exclusive or necessarily predominant secondary metabolites of *P. americana* [152]. Esculentoside P (Figure 6(**2**)), in particular, has been identified as a major bioactive phytolaccasaponin in fractions obtained from *P. americana* roots.

Americanin A (Figure 6(**3**)), a previously known neolignan occurring in the seeds of *P. americana*, was isolated and subsequently investigated for its biological activity by Jung et al. [153]. The species has also been reported to contain flavonoids such as quercetin, kaempferol, and isoquercitrin [151]. In addition, the phytosterol α-spinasterol (Figure 6(**4**)) was isolated from a chloroform extract of the roots following activity-guided fractionation [154].

Extracts and isolated constituents obtained from species of the genus *Phytolacca*, including *P. americana*, have been investigated in a range of preclinical models and have shown anti-inflammatory, antiparasitic, antiviral, and antiproliferative activities [151]. Nevertheless, these findings are derived mainly from in vitro experiments and animal studies and should not be interpreted as evidence of established clinical efficacy.

Americanin A was evaluated for its antiproliferative and antitumor effects against HCT116 human colorectal carcinoma cells. In vitro, the compound inhibited HCT116 cell proliferation in a concentration- and time-dependent manner and induced G2/M cell-cycle arrest through modulation of the ATM/ATR signaling pathway and the Skp2–p27 axis. In a xenograft model, intraperitoneal administration of americanin A reduced the growth of HCT116 tumors in nude mice without producing overt toxicity under the experimental conditions employed. Irinotecan was included as a positive control; however, the results should be presented as evidence of preclinical antitumor activity rather than as proof of therapeutic equivalence or superiority to irinotecan [153].

Pokeweed antiviral protein, commonly abbreviated as PAP, is a ribosome-inactivating protein with RNA N-glycosidase activity isolated from *P. americana*. PAP inhibited Japanese encephalitis virus replication in vitro in a concentration-dependent manner, with an IC_50_ of 300 ng/mL, corresponding to approximately 23.1 nM. In mice challenged with a lethal dose of the virus, intraperitoneal administration of PAP at 1 mg/kg increased survival when administered either before or after infection. Survival rates of 87.5% and 85.7% were reported for pre-infection and post-infection administration, respectively [155]. These results support the experimental antiviral activity of PAP, although its ribosome-inactivating properties as well as potential toxicity remain important limitations to its direct therapeutic application.

The phytosterol α-spinasterol, isolated from the chloroform fraction of *P. americana* roots, potently inhibited high-glucose-induced proliferation of glomerular mesangial cells, with an IC_50_ of approximately 9.5 pmol/L. In this experimental system, its inhibitory potency was reported to be approximately 1000-fold greater than that of simvastatin, which was used as the positive control. In streptozotocin-induced diabetic mice, α-spinasterol also reduced serum triglyceride levels, renal weight, and urinary protein excretion. These results indicate that the compound may modulate selected processes associated with diabetic nephropathy, but they do not establish its efficacy or safety as a treatment in humans [154].

The toxicity of *P. americana* is due to triterpenoid saponins [156] and is clinically relevant. Woolum et al. [157] retrospectively analyzed 1669 human exposures reported to the Kentucky Poison Control Center between 2000 and 2019. Oral ingestion accounted for 98.3% of the reported exposures, and berries were involved in 93.9% of cases of ingestion. Although the exposures were generally well tolerated, abdominal pain, nausea, vomiting, and diarrhea were the most frequently recorded adverse effects. Dermal exposure was associated with cutaneous edema, pain, and swelling. Treatment was mainly supportive, and no deaths were reported during the study period. This data should not, however, be interpreted as evidence that the plant is safe for consumption, since toxicity may vary according to the plant part, quantity ingested, and individual vulnerability [157]. Widespread 19th-century poisonings in eastern North America resulted from overdosing on poke-root tinctures used as antirheumatics and accidentally ingesting pokeweed berries or roots mistaken for parsnip. Recoveries were documented more often than fatalities and yet the plant should be used with caution [156]. Two people reached the emergency room (no symptoms were described in this paper) after consumption of the plant root and the analyses revealed 1.9 ng/mL of esculentoside A, 1.5 ng/mL of esculentoside C, and esculentoside B and H below the LLOQ in the blood sample [156]. Importantly, a fatal case reported in China in 2025 involved the ingestion of approximately 150 g of raw-equivalent *P. americana* leaves that had been stir-fried, demonstrating that cooking described as a culinary preparation does not necessarily eliminate toxicity [158]. The case highlights the lethal potential of improperly prepared pokeweed leaves and the need to distinguish traditional food use from demonstrated toxicological safety. So far, no universally safe dose has been specified due to the absence of validated dose–response data. The effect of cooking requires especially cautious interpretation. Although boiling has been reported to reduce the toxicity of some pokeweed constituents, the available clinical literature does not establish a validated household procedure that consistently renders the plant safe. Overall, the literature does not establish a reliable safe-consumption threshold for *P. americana*. The approximately 150 g raw-equivalent amount in the fatal case is a case-specific exposure—not a toxic-dose threshold—and should not be used to infer a safe or unsafe cutoff. The evidence also supports caution about cooking, rather than a claim that any particular cooking method guarantees detoxification. Interestingly the aboveground parts of the plant are reported nontoxic early in the spring [156], suggesting that toxic compounds accumulate after that period, therefore a metabolomic analysis is necessary to definitively confirm these dynamics.

#### 2.3.6. *Carpobrotus edulis*

**Figure 7:**
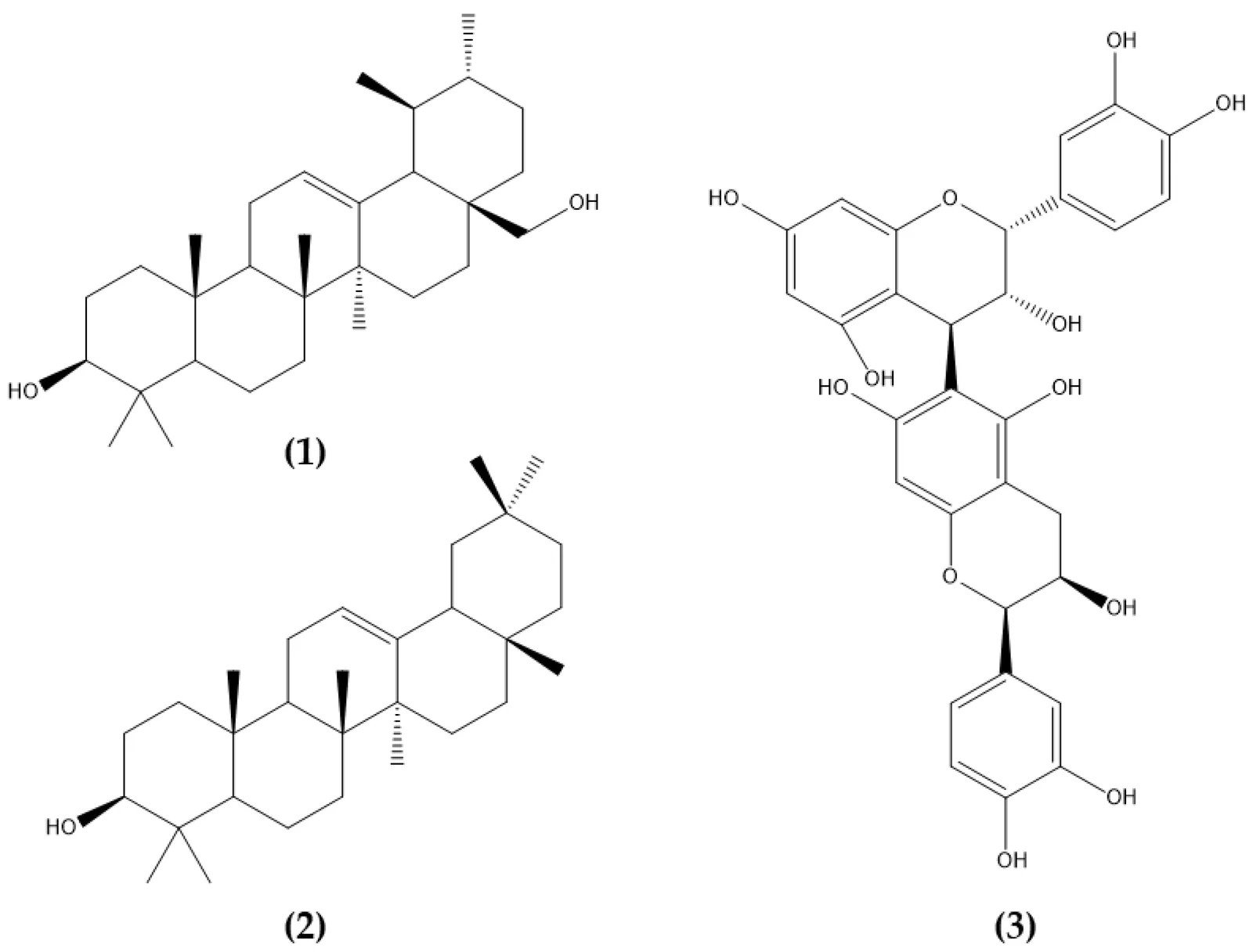
Notable phytochemicals in *C. edulis*. Uvaol (**1**), *β*-amyrin (**2**), and Procyanidin B5 (**3**).

Phytochemical profiling of extracts obtained from the peels and flesh of mature *C. edulis* fruits was performed using high-performance liquid chromatography coupled with electrospray ionization tandem mass spectrometry. Water, ethanol, and acetone were used as extraction solvents. The identified constituents belonged predominantly to the phenolic acid, flavonoid, and coumarin classes, with several flavonoids occurring as mono-, di-, or tri-*O*-glycosylated derivatives. Peel extracts generally displayed a greater chemical diversity than flesh extracts, and uvaol (Figure 7), an ursane-type pentacyclic triterpenoid, was among the constituents detected in the peel [159].

Further phytochemical investigation was carried out through bioassay-guided fractionation of a methanolic extract of *C. edulis*. Separation of its chloroform- and ethyl acetate-soluble fractions led to the isolation of seven compounds: β-amyrin, oleanolic acid, uvaol, monogalactosyldiacylglycerol, catechin, epicatechin, and procyanidin B5 (Figure 7). Their structures were established using mass spectrometry together with one- and two-dimensional NMR spectroscopy [160].

Another study characterized the polyphenolic profile of an aqueous–acetone extract of *C. edulis* using UPLC-DAD. Chlorogenic acid was identified as the predominant compound, accounting for 43.7% of the detected polyphenolic constituents [161]. This evidence supports the relevance of chlorogenic acid in the phytochemical profile of the species; however, the absolute concentration of 107 ± 10 µg/g reported in the study could not be confirmed from the accessible information and should be verified against the complete analytical data before inclusion.

Extracts and isolated constituents of *C. edulis* have been investigated in several preclinical models for antimicrobial, antifungal, neuroprotective, enzyme-inhibitory, and antiproliferative properties. Nevertheless, the available findings derive mainly from in vitro experiments and should therefore be interpreted as preliminary evidence of biological activity rather than proof of therapeutic efficacy [26]. The neuroprotective potential of an aqueous *C. edulis* extract was evaluated in SH-SY5Y human dopaminergic cells exposed to 1-methyl-4-phenylpyridinium, or MPP^+^, as an in vitro model of Parkinsonian neurotoxicity. Pretreatment with the extract attenuated the MPP^+^-induced reduction in cell viability, intracellular reactive oxygen species accumulation, nuclear condensation, and activation of caspases 9 and 3/7. These results suggest antioxidant and anti-apoptotic effects in this cellular model but do not demonstrate efficacy in animal models or patients with Parkinson’s disease [162].

The α-glucosidase inhibitory activity of aqueous, 50% methanolic, and 70% acetone extracts prepared from *C. edulis* leaves was also examined in vitro. The aqueous extract showed the strongest inhibitory effect, with an IC_50_ value of 5 µg/mL. In contrast, acarbose, used as the positive control, showed an IC_50_ of 429 µg/mL under the same experimental conditions [163]. Although this marked difference indicates strong enzyme inhibition by the aqueous extract, it cannot be directly interpreted as evidence that the plant preparation is more effective than acarbose in vivo, because extract composition, bioavailability, metabolism, and safety were not evaluated in this enzymatic assay.

The seven compounds isolated by Martins et al. were evaluated in a multidrug-resistant mouse lymphoma cell model expressing the human MDR1 gene. The study focused primarily on their ability to inhibit P-glycoprotein, the membrane efflux transporter involved in multidrug resistance. Among the isolated constituents, uvaol showed the greatest ability to reverse the resistant phenotype. This finding is more accurately described as P-glycoprotein inhibition and multidrug-resistance reversal rather than as a direct antiproliferative effect [160].

The preliminary toxicity of aqueous and methanolic leaf extracts was assessed using a modified *Artemia franciscana* nauplii lethality assay. The *C. edulis* extracts did not induce mortality significantly above that observed in the negative control. They were consequently classified as non-toxic or of low toxicity within this experimental model [164]. However, the *Artemia* assay is intended as an initial general-toxicity screen and cannot replace toxicological studies in mammalian cells, experimental animals, or humans.

**Table 2:** An overview of the bioactive compounds, pharmacological activity and toxicity of the species.

Plant Species	Main Bioactive Compounds	Reported Pharmacological Effects	Toxicological Evidence	Refs.
*Amaranthus hybridus*	Polyphenols and flavonoids; alkaloids; saponins; cardenolides; polysaccharides	Antidiabetic and antioxidant effects; hepatoprotective activity (preclinical)	Low toxicity in the brine-shrimp assay (LC_50_ = 6233.6 µg/mL); nephrotoxicosis reported in cattle following substantial consumption	[22,126,130,165]
*Reynoutria × bohemica*	Stilbenes (resveratrol, polydatin); anthraquinones (emodin); flavonoids; flavan-3-ols and procyanidins; hydroxycinnamic acids; carotenoids	α-amylase inhibition; cellular antioxidant activity; non-selective cytotoxicity and antiproliferative effects in vitro	No dedicated toxicological assessment was identified in the cited studies; human safety remains undetermined	[131,132,133,134,135,136]
*Opuntia humifusa*	Phenolic acids (ferulic, protocatechuic and *p*-coumaric acids); flavonoids (taxifolin, myricetin); other phenolic constituents	Antioxidant and anti-inflammatory activity; Antiproliferative effects in U87MG cells; potential antidiabetic effects (preclinical)	No treatment-related adverse effects up to 2000 mg/kg in single- and 14-day oral rat studies; chronic and human safety not established	[40,89,122,139,140,141]
*Oxalis corniculata*	Flavone and flavonol glycosides; alkaloids; lignans; organic acids; terpenoids; coumarins; polysaccharides; anthraquinones	Anti-inflammatory activity; α-glucosidase inhibition and postprandial glucose-lowering effects; cytotoxic/antiproliferative, hepato- and neuroprotective effects (preclinical)	No overt acute toxicity up to 2000 mg/kg in mice; calcium oxalate content warrants caution in individuals predisposed to nephrolithiasis	[142,143,144,145,146,147,148,149,150]
*Phytolacca americana*	Esculentosides and other oleanane-type triterpenoid saponins; lignans/neolignans; flavonoids; phytosterols (α-spinasterol); pokeweed antiviral protein (PAP)	Anti-inflammatory and antiparasitic effects; antiproliferative/antitumour activity; antiviral activity; inhibition of high-glucose-induced mesangial-cell proliferation (preclinical)	Oral exposure may cause abdominal pain, nausea, vomiting and diarrhea; dermal exposure may cause edema, pain and swelling; no deaths were reported in the observational series	[151,152,153,154,155,157]
*Carpobrotus edulis*	Phenolic acids (including chlorogenic acid); mono-, di- and tri-*O*-glycosylated flavonoids; coumarins; triterpenoids (uvaol, β-amyrin, oleanolic acid); catechin, epicatechin and procyanidin B5	Antimicrobial and antifungal activity; neuroprotective effects; α-glucosidase inhibition; P-glycoprotein inhibition and multidrug-resistance reversal (preclinical)	No mortality significantly above control in the Artemia franciscana assay; mammalian and human safety remain undetermined	[111,159,160,161,162,163,164]

Distinguishing between plant parts traditionally consumed as food and those investigated in phytochemical and pharmacological studies is essential for a clear and meaningful interpretation of the evidence (Table 3). Traditional food use reflects established practices of human consumption, whereas phytochemical and pharmacological studies may examine different plant organs, extracts, or isolated compounds that may not be consumed traditionally. These distinctions are particularly important when assessing the potential contribution of invasive alien plants to food-related ecosystem services and their possible value as biological resources.

The overall evidence level of the reported pharmacological effects (Table 4) can be summarized as follows: *A. hybridus*—Preclinical (in vitro and in vivo); species-specific evidence remains limited; *R.* × *bohemica*—Preclinical (in vitro only); *O. humifusa*—Preclinical (in vitro and in vivo); findings are preliminary; *O. corniculata*—Preclinical (in vitro and in vivo); *P. americana*—Preclinical (in vitro and in vivo); no clinical efficacy evidence; *C. edulis*—Preclinical (predominantly in vitro)

## 3. Materials and Methods

We accessed Google Scholar, Web of Science, and PubMed to identify publications for the period 1990–2026. The literature search was conducted in the period between December 2025 and August 2026. The search strategy included the following plant taxa: *Amaranthus hybridus*, *Reynoutria × bohemica* (also referred to as *Fallopia × bohemica*), *Reynoutria japonica* (also referred to as *Fallopia japonica*), *Opuntia humifusa*, *Oxalis corniculata*, *Phytolacca americana*, and *Carpobrotus edulis*. These taxon names were combined with keywords related to the chemical composition and biological properties of the plants, including “compounds,” “metabolites,” “effects,” “activity,” and “toxicity.”

The nomenclature of the taxa cited in the text follows POWO (2026) [28].

**Figure 8:**
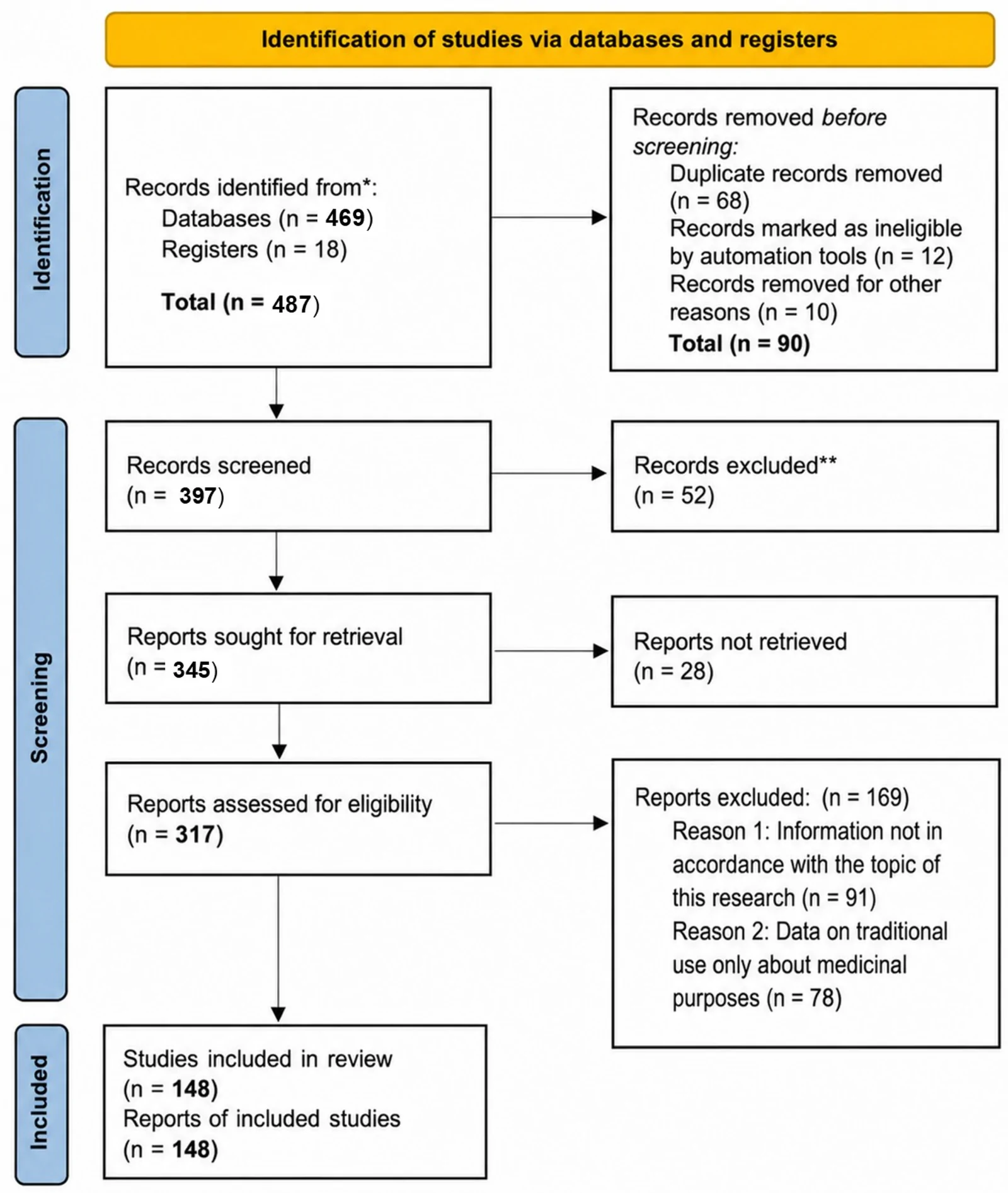
PRISMA protocol flow chart (the introductory citations are not included here). * total number of publications, ** not relevant.

Publications were included if they were relevant to the objectives of the review and provided information on the chemical constituents, metabolites, biological activities, effects, toxicity, or traditional uses of the selected plant taxa, as applicable to the scope of the review. Publications addressing traditional uses exclusively for medicinal purposes were excluded, as were publications that did not contain information relevant to the research topic. Records were screened for eligibility in accordance with the PRISMA 2020 guidelines [166]. In total, 169 papers were excluded based on these criteria. The study selection process is summarized in Figure 8, which presents the PRISMA 2020 flow diagram illustrating the identification, screening, eligibility assessment, and selection of publications included in the review. The references for the databases consulted during the literature search are provided [167,168,169].

## 4. Conclusions

Edible alien invasive plants represent a complex and underexplored biological resource that may provide opportunities for both biodiversity management and the discovery of bioactive compounds. This comprehensive review highlights six invasive taxa—*Amaranthus hybridus*, *Reynoutria × bohemica*, *Opuntia humifusa*, *Oxalis corniculata*, *Phytolacca americana*, and *Carpobrotus edulis*—that combine documented or traditional food uses with diverse phytochemical profiles and reported pharmacological activities.

The reviewed species contain chemically diverse secondary metabolites, including phenolic acids, flavonoids, stilbenes, anthraquinones, terpenoids, alkaloids, and saponins, which have been associated with antioxidant, anti-inflammatory, antidiabetic, antimicrobial, hepatoprotective, neuroprotective, and antiproliferative effects in preclinical studies. In particular, *Reynoutria × bohemica* is characterized by a complex phytochemical profile including stilbenes, anthraquinones, flavonoids, and hydroxycinnamic acids, with resveratrol, epicatechin, emodin, polydatin, catechin derivatives, and procyanidin B2 among the compounds reported from its rhizomes.

At the same time, the potential utilization of invasive plants should not be interpreted as evidence of established therapeutic or food safety. Important species-specific risks are documented, including calcium oxalate accumulation in *O. corniculata* and triterpene saponin-associated toxicity in *P. americana*, while the available safety information for several other species remains limited. For *R. × bohemica*, no specific toxicity has been systematically established in the reviewed literature, although some extracts have produced basic cytotoxic effects in experimental assays.

Therefore, the valorization of edible invasive plants should be considered within a risk-aware and evidence-based framework, rather than as an alternative to conventional invasive species control. Future research should prioritize standardized phytochemical characterization, assessment of geographical and seasonal variation, comprehensive toxicological evaluation, identification of active compounds and mechanisms of action, and well-designed in vivo and clinical studies. Such evidence could determine whether selected invasive species can be safely developed as sources of food ingredients, nutraceuticals, or bioactive compounds while simultaneously contributing to sustainable biomass management. For example, important objectives for further research are to establish the toxic-dose threshold for *P. americana* and which cooking method guarantees detoxification.

Overall, transforming problematic invasive biomass into scientifically validated bioresources represents a promising approach that requires careful integration of ecological, ethnobotanical, pharmacological, and toxicological evidence.

## Figures and Tables

**Table 3:** Traditionally consumed plant parts and plant materials used in phytochemical and pharmacological investigations of selected edible invasive alien plants.

Plant Species	Plant Parts Traditionally Used for Food	Plant Parts Used in Phytochemical Studies	Plant Parts Used in Pharmacological Tests
*Amaranthus hybridus*	Leaves, young shoots/stems, seeds, inflorescences	Aerial parts, leaves	Extracts/fractions from Aerial parts, leaves, Polysaccharides isolated from aerial parts
*Reynoutria × bohemica*	Young shoots, stems, leaves (traditional uses documented mainly for the progenitor *R. japonica*)	Rhizomes, leaves	Extracts/fractions from rhizomes, flowers
*Opuntia humifusa*	Young stems (cladodes), fruits	different plant parts (not always specified)	Extracts/fractions from different plant parts (not always specified)
*Oxalis corniculata*	Leaves	Aerial parts, leaves	Whole plant, aerial parts/plant extracts (part not specified in some studies)
*Phytolacca americana*	Young shoots, leaves, stems, peeled stalks; berries used in beverages and as a food colorant	Roots, seeds	Seeds, roots; pokeweed antiviral protein (plant part not specified in the reviewed study)
*Carpobrotus edulis*	Fruits, leaves	Fruit peels, fruit flesh, whole-plant material	Leaves, whole-plant extracts, fruit-derived extracts/constituents

**Table 4:** Evidence levels of the reported pharmacological effects of the reviewed invasive alien plant species.

Plant Species	In Vitro Evidence	In Vivo Evidence	Clinical Evidence
*Amaranthus hybridus*	Glucose uptake in L6 myoblasts and C3A hepatocytes; antioxidant activity of polysaccharides; α-glucosidase inhibition reported in multi-species studies	Dietary supplementation associated with reduced heavy metal-induced tissue damage in animal models	No clinical evidence of therapeutic efficacy identified in the review
*Reynoutria × bohemica*	α-Amylase inhibition, antiglycoxidative and cellular antioxidant activities; non-selective cytotoxicity in cancer and non-tumor cell lines	No in vivo pharmacological evidence described	No clinical evidence identified
*Opuntia humifusa*	Antiproliferative effects in U87MG glioblastoma cells; antioxidant and anti-inflammatory activities; enzyme inhibition	Improvements in glucose- and lipid-related parameters reported in diabetic animal models; short-term oral toxicity evaluated in rats	No clinical evidence of therapeutic efficacy identified in the review
*Oxalis corniculata*	Anti-inflammatory activity; cytotoxic/antiproliferative effects; α-glucosidase inhibition	Reduced postprandial blood glucose reported in mice; acute toxicity evaluated in mice	No clinical evidence of therapeutic efficacy identified
*Phytolacca americana*	Antiproliferative activity of americanin A; inhibition of mesangial-cell proliferation; antiviral activity of PAP	Antitumor effects in a mouse xenograft model; antiviral effects of PAP in infected mice; α-spinasterol-associated effects in diabetic mice	Human poison-control data describe exposures and adverse effects, not therapeutic efficacy
*Carpobrotus edulis*	Neuroprotective effects in SH-SY5Y cells; α-glucosidase inhibition; P-glycoprotein inhibition and multidrug-resistance reversal; antimicrobial and antifungal activities reported	No in vivo pharmacological efficacy evidence described in the review	No clinical evidence of therapeutic efficacy identified

## Data Availability

Data connected with this study are freely available from the corresponding author, upon reasonable written request.

## Abbreviations

The following abbreviations are used in this manuscript:

CERNUT	Research Centre on Nutraceuticals and Health Products
COX-2	Cyclooxygenase-2
DOAJ	Directory of Open Access Journals
DPPH	2,2-Diphenyl-1-picrylhydrazyl
GC-MS	Gas Chromatography–Mass Spectrometry
HEK293T	Human Embryonic Kidney 293T
HepG2	Hepatocellular Carcinoma Human Cell Line
HPLC-DAD	High-Performance Liquid Chromatography with Diode-Array Detection
iNOS	Inducible Nitric Oxide Synthase
LC_50_	Lethal Concentration 50%
MTT	3-(4,5-Dimethylthiazol-2-yl)-2,5-diphenyltetrazolium bromide
PaTu	Pancreatic Tumor Cell Line
PAP	Pokeweed Antiviral Protein
RAW264.7	Murine Macrophage Cell Line
TLC	Thin-Layer Chromatography
TNF-α	Tumor Necrosis Factor-Alpha
U87MG	Human Glioblastoma Cell Line
